# Progress Test in nursing: perspectives for undergraduate education

**DOI:** 10.1590/0034-7167-2023-0413

**Published:** 2024-09-06

**Authors:** Bruna Moreno Dias, Lúcia Marta Giunta da Silva, Marina de Góes Salvetti, Carmen Silvia Gabriel

**Affiliations:** I Universidade de São Paulo. Ribeirão Preto, São Paulo, Brazil; II Universidade Federal de São Paulo. São Paulo, São Paulo, Brazil; III Universidade de São Paulo. São Paulo, São Paulo, Brazil

**Keywords:** Education, Nursing, Educational Measurement, Academic Performance, Nursing Faculty Practice, Schools, Educación en Enfermería, Evaluación Educacional, Rendimiento Académico, Práctica del Docente de Enfermería, Instituciones Académicas

## Abstract

**Objective:**

to reflect on the perspectives of adopting the Progress Test in undergraduate nursing education.

**Methods:**

this is a reflective study, based on authors’ critical thinking and supported by national and international literature on the Progress Test application in undergraduate health courses.

**Results:**

the Progress Test is as a valuable teaching-learning strategy, with potential applications and benefits for students, professors, and academic management.

**Final considerations:**

systematic nursing education assessment indicates valuable information for different stakeholders. Understanding its potential benefits, the Progress Test is presented as a strategy that can be replicated in undergraduate nursing education, either individually, by institutions, or collaboratively, by the establishment of partnerships or consortiums of institutions.

## RESUMEN

**Objetivo:**

reflexionar sobre las perspectivas de adopción de la Prueba de Progreso en la formación de pregrado en enfermería.

**Métodos:**

se trata de un estudio reflexivo, basado en el pensamiento crítico de las autoras y sustentado en literatura nacional e internacional sobre la aplicación de la Prueba de Progreso en cursos de pregrado en el área de la salud.

**Resultados:**

la Prueba de Progreso se presenta como una valiosa estrategia de enseñanza-aprendizaje, con potenciales aplicaciones y benefícios para estudiantes, docentes y gestión académica.

**Consideraciones finales:**

la evaluación sistemática de la educación en enfermería indica información valiosa para diferentes actores. Entendiendo sus potenciales beneficios, la Prueba de Progreso se presenta como una estrategia que puede ser replicada en la educación de pregrado en enfermería, sea de manera individual, por instituciones, o de manera colaborativa, a través del establecimiento de alianzas o consorcios de instituciones.

## INTRODUCTION

Nursing education is one of the priority areas of the World Health Organization’s (WHO) Global Strategic Directions for Nursing and Midwifery 2021-2025, due to the positive impact of investment actions in nursing education and potential contributions of these professionals to healthcare services and systems. Among the strategic actions proposed by the WHO is the adoption of different learning and teaching assessment strategies, in order to guarantee quality education and professional training^(1)^.

Nationally, assessing the teaching-learning process of undergraduate nursing courses is essential given the expansion of the offer of vacancies and undergraduate nursing courses and the controversies surrounding quality training of students from these courses^(2)^.

Among the possible actions and initiatives adopted to assess undergraduate nursing education, the Progress Test (PT) stands out, understood as a systematic and longitudinal assessment of student performance, capable of being applied in different time intervals, formats and assessment levels. Through PT, it is possible to carry out cognitive assessment and verification of students’ knowledge gain in a continuous and progressive manner throughout the course^(3)^.

Even though it is recognized that PT should not be the only form of knowledge assessment, there is evidence that its development, application and analysis of results offer benefits in terms of assessing learning and improving the pedagogical program and the teaching-learning process, with valuable information for students, professors and educational institutions, as explored in this reflection^(4)^.

In health education, PT has been widely used in undergraduate medical courses in Brazil and other European countries since the 1980s^(5)^. In nursing, PT application is relatively new, having been carried out at first through isolated initiatives, by *Universidade Federal de São Paulo Escola Paulista de Enfermagem* (EPE-UNIFESP) and *Universidade de São Paulo Escola de Enfermagem de Ribeirão Preto* (EERP-USP), and, subsequently, through unified initiative, by the São Paulo Nursing Courses Consortium for PT, since 2019. In addition to these initiatives, no other national or international experiences of applying PT in nursing courses were identified.

Given the innovative nature of the proposal for education and potential benefits of PT for students, professors, and academic institutions, it is proposed to reflect on the unified application of the test in undergraduate nursing courses with a view to rethinking training assessment strategies and encouraging the adoption of new pedagogical practices.

## OBJECTIVE

To reflect on the perspectives of adopting PT in undergraduate nursing education.

## METHODS

This is a reflective study, based on authors’ critical thinking and supported by national and international literature on PT application, taking into account the perspectives of students, professors and academic management.

Considering the novelty of this initiative and the lack of identification of national or international literature on test application in undergraduate nursing courses, this reflection is based on available literature on PT application in undergraduate health courses.

This reflection is based on the experience of applying PT in Public Schools of Nursing in São Paulo, applied by the São Paulo Nursing Courses Consortium, composed of eight public educational institutions in the state of São Paulo as: EERP-USP; *Escola de Enfermagem da USP* (EE-USP); EPE-UNIFESP; *Faculdade de Medicina de Marília* (FAMEMA); *Faculdade de Medicina de São José do Rio Preto* (FAMERP); *Universidade Estadual Paulista Faculdade de Medicina de Botucatu* (UNESP); *Universidade de Campinas* (UNICAMP) School of Nursing; and *Universidade Federal de São Carlos* (UFSCar).

The test is administered annually, consisting of 120 objective questions, distributed across six areas of knowledge (20 questions on public health, 15 on management, 20 on child and adolescent health, 20 on women’s health, 35 on adult health and ten on mental health), with assessment of student performance measured by the number of correct answers and by a grade on a scale of 0 to 10 points per area and total.

Question preparation, review and selection are part of a process carried out annually, according to the list of themes defined by the consortium and its own schedule. The questions are prepared by professors from participating institutions and reviewed and collectively selected by expert professors in the areas, representatives of all participating institutions. Among the 120 questions in the test, 35 are pre-tested questions, which are previously applied questions, distributed evenly across the six areas of knowledge, which presented excellent or good psychometric performance, whereas the remaining 85 questions are new, prepared exclusively for the test of that year.

All students from all years of the eight educational institutions are eligible to take and are encouraged to participate in the test, which has operational costs fully covered by the institutions, without payment of any fee by students, who participate in test voluntarily.

## REFLECTIONS ABOUT THE PROGRESS TEST IN NURSING

The reflections presented below are structured from the perspectives of students, professors and academic management.

### Student

Experiences of applying PT in medical education point to three main benefits for students: academic learning assessment; support in the learning process; and preparation for licensing exams^(6)^.

For students, PT makes it possible to periodically assess their progress throughout the course, with reflection on the knowledge obtained at each stage of the course, resulting in self-responsibility for their learning. After taking the test, students can analyze their performance through the score obtained (total and by area), comparison of their individual performance with other students in their year from all institutions and with other students in their institution and magnitude of individual student progression in relation to performance in tests from previous years, when applicable, making it possible to reflect on their degree of cognitive mastery in areas covered in the test^(5)^.

From the perspective of training feedback, in addition to performance analysis, students have access to the test with a commented answer sheet and references. For each test question, comments are presented about correct and incorrect items and references that support themes and concepts covered in each question. In this regard, through self-management and critical thinking, the test allows students to identify their strengths and weaknesses and identify opportunities to improve their knowledge.

PT, through its feedback based on commented answer sheets and references, offers students an opportunity to learn about themes and concepts expected for each area of knowledge, making it possible for students to seek to improve their academic performance and advance their learning on various topics covered in the test^(3, 5)^.

Such contributions are supported by the opinion of undergraduate medical students who, when interviewed about their participation in PT, considered the test a relevant “pedagogical tool”, which allows self-assessment and correction of learning gaps^(7)^.

Finally, PT provides an opportunity for students to prepare to take other exams with a similar structure, such as competitive exams or residency selection processes. Even though it is not a reality in Brazil, PT makes it possible to prepare for professional licensing tests, like the experience of other countries, such as the United States of America.

### Professor

Assessing knowledge is an important activity in teaching. For professors, PT allows reflection on content covered in subjects and knowledge expected to be acquired by students. Based on student performance results, professors are able to carry out a diagnosis of teaching and the implementation of educational interventions and continuous improvement actions for the course, in addition to identifying opportunities for professor development.

PT can be used in a complementary way to the traditional model of assessment of subjects taught by professors, allowing broader analyzes of student and/or class performance, generating less tension between professors and students when compared to traditional assessment instruments aimed at student approval/failure^(4)^.

By working on question preparation, review and selection, professors can improve their skills in developing objective questions for student assessment, considering good practices for preparing multiple-choice questions. Additionally, it is important to highlight the relevance of initiatives, individual or institutional, for professors to develop the ability to develop such questions, as they must ensure that assessment results are valid and reliable.

By working together with colleagues from other areas and other institutions, professors can broaden the reflection on the profile of knowledge necessary for nursing training, analyzing professional practice from the perspective of different professors and institutions.

In this regard, in addition to support in preparing questions, professors need feedback on the good quality of items, considering student performance and psychometric assessment of questions.^(8)^

### Academic management

From the perspective of academic management, it is possible to identify topics with higher or lower performance, disaggregated by undergraduate year and area of knowledge. Thus, given the possibility of identifying training gaps, it is possible to propose and implement improvement actions for the course, such as adapting the pedagogical program and proposing teaching-learning strategies in order to guarantee knowledge acquisition, with a view to improving teaching.

Institutional performance analysis allows inferences to be made about the effectiveness of the course curriculum, identifying its strengths and vulnerabilities, with the possibility of reviewing it or even verifying the impact of curricular changes made^(5)^.

Thus, decision-makers, such as course coordinators in teaching areas and representatives of collegiate bodies, can use assessment to review the pedagogical program and the contents covered, in addition to proposing changes in subjects, areas of knowledge or in the academic program and/or course curriculum^(3)^.

Such applications have been reported in the education experience in medical schools, in which PT has been used to assess curricular changes, compare traditional curricula and curricula with active learning methodologies, and assist in developing new curricula^(4, 6)^.

It is also possible to carry out benchmarking disaggregated by area of knowledge and undergraduate year between participating institutions, allowing contextualized analyses, without ranking, of different pedagogical strategies and curricular models^(6)^.

In addition to the more comprehensive actions, through individual student performance analysis, it is possible to identify students with high and low performance and outline personalized pedagogical support actions for each case^(6)^.

Considering the above, institutions must take an active role in PT operationalization, encouraging and favoring the involvement and active participation of students and professors.

From an institutional perspective, it is important to invest in measures that encourage the active participation of all students as well as to encourage spaces for reflection and discussion of performance between students and professors, favoring the use of PT results as a learning strategy^(5)^.

Institutions must promote professor development actions to prepare objective questions, with strategies such as workshops, technical guidance, and spaces for discussion on the quality of questions and analysis of results^(3)^.

### Challenges and limitations

Although there are benefits to using PT, its limitations and challenges must be considered. From an operational perspective, organizing and carrying out PT are arduous tasks that involve different stakeholders and institutions and establishing a schedule that suits the institutional agenda and the implication of financial resources^(4)^.

It is necessary to encourage discussions between the players involved and be clear about the objectives of the test, themes and areas of interest, aligned with competencies required of nurses as well as strategies for analyzing and using the results in the teaching-learning process^(7)^.

Aiming at the test quality and validity, teaching staff training to prepare objective questions must be considered in different and continuous strategies^(9)^.

Given students’ voluntary participation, it is possible to infer that participating students are those with greater involvement and performance in the course. Therefore, it is important to encourage the broad participation of all students, in order to obtain results that represent the institution as a whole and, consequently, contribute to actions to improve education^(10)^.

Even if it is considered that PT enables formative feedback, it must be considered that self-assessment practice does not yet constitute culture in academic life, and it is necessary to propose spaces for reflection and discussion of issues and individual, class and course results^(7)^.

Finally, it is worth highlighting that PT provides performance assessment at the cognitive level, making it possible for students to obtain a high score on the test, but present deficiencies in mastering other skills and attitudes^(3)^. Therefore, although PT is an important tool, it needs to be applied in an integrated manner with other assessment strategies beyond the cognitive level.

## FINAL CONSIDERATIONS

PT adoption is a strategy for assessing undergraduate nursing education, adding to the actions to qualify the teaching-learning process.

Systematic nursing education assessment, through PT, allows the provision of valuable information for different stakeholders. Considering the potential benefits of the test for students, professors and academic management, PT is presented as a strategy that can be replicated in undergraduate nursing education, whether individually, by institutions, or collaboratively, by the establishment of partnerships and consortia between institutions.
