# Supplementary material for: Progress Test in nursing: perspectives for undergraduate education
Source: Rev Bras Enferm. 2024 Sep 6;77(4):e20230413. doi: 10.1590/0034-7167-2023-0413 (PMC11382674; doi:10.1590/0034-7167-2023-0413)
Supplement: Translation

# Translation: Teste de Progresso na enfermagem: perspectivas para o ensino de graduação

**DOI:** 10.1590/0034-7167-2023-0413pt

**Authors:** Bruna Moreno Dias, Lúcia Marta Giunta da Silva, Marina de Góes Salvetti, Carmen Silvia Gabriel

**Affiliations:** I Universidade de São Paulo. Ribeirão Preto, São Paulo, Brasil; II Universidade Federal de São Paulo. São Paulo, São Paulo, Brasil; III Universidade de São Paulo. São Paulo, São Paulo, Brasil

**Keywords:** Educação em Enfermagem, Avaliação Educacional, Desempenho Acadêmico, Prática do Docente de Enfermagem, Instituições Acadêmicas

## Abstract

**Objetivo:**

refletir acerca das perspectivas da adoção do Teste de Progresso no ensino de graduação em enfermagem.

**Métodos:**

trata-se de estudo reflexivo, baseado no pensamento crítico das autoras e sustentado na literatura nacional e internacional sobre a aplicação do teste de progresso em cursos de graduação na área da saúde.

**Resultados:**

o teste de progresso se apresenta como uma valiosa estratégia de ensino-aprendizagem, com potenciais aplicações e benefícios para estudantes, docentes e gestão acadêmica.

**Considerações finais:**

a avaliação sistemática do ensino de enfermagem indica informações valiosas para diferentes atores. Entendendo seus potenciais benefícios, apresenta-se o Teste de Progresso como estratégia passível de ser replicada no ensino de graduação em enfermagem, seja de forma individual, pelas instituições, ou de maneira colaborativa, por meio do estabelecimento de parcerias ou consórcios de instituições.

## INTRODUÇÃO

O ensino é uma das áreas prioritárias das Orientações Estratégicas Mundiais para Enfermagem e Obstetrícia 2021-2025 da Organização Mundial da Saúde (OMS), em razão do impacto positivo das ações de investimento no ensino de enfermagem e potenciais contribuições desses profissionais aos serviços e sistemas de saúde. Entre as ações estratégicas propostas pela OMS, está a adoção de diferentes estratégias de ensino e de avaliação do ensino, no sentido de garantir a qualidade da formação e capacitação profissional^(1)^.

No contexto nacional, a avaliação do processo de ensino-aprendizagem dos cursos de graduação em enfermagem é essencial diante da expansão da oferta de vagas e cursos de graduação em enfermagem e das controvérsias em torno da qualidade da formação de egressos desses cursos^(2)^.

Entre as possíveis ações e iniciativas adotadas para a avaliação do ensino de graduação em enfermagem, aponta-se o Teste de Progresso (TP), entendido como uma avaliação sistemática e longitudinal do desempenho dos estudantes, passível de ser aplicado em diferentes intervalos de tempo, formatos e níveis de avaliação. Por meio do TP, é possível realizar a avaliação cognitiva e a verificação do ganho de conhecimento do estudante de forma contínua e progressiva ao longo de toda a graduação^(3)^.

Ainda que se reconheça que o TP não deva ser a única forma de avaliação do conhecimento, há evidências de que seu desenvolvimento, aplicação e análise de resultados oferecem benefícios em termos de avaliação do aprendizado e de melhorias do projeto pedagógico e do processo de ensino-aprendizagem, com informações valiosas para estudantes, docentes e instituições de ensino, tal como explorado nesta reflexão^(4)^.

Na formação em saúde, o TP tem sido amplamente utilizado pelos cursos de graduação em medicina no Brasil e em outros países da Europa desde a década de 1980^(5)^. Na enfermagem, a aplicação do TP é incipiente, tendo sido realizada, a princípio, por meio de iniciativas isoladas, pela Escola Paulista de Enfermagem da Universidade Federal de São Paulo (EPE-UNIFESP) e Escola de Enfermagem de Ribeirão Preto da Universidade de São Paulo (EERP-USP), e, posteriormente, por meio de iniciativa unificada, pelo Consórcio dos Cursos de Enfermagem Paulistas para o TP, desde o ano de 2019. Além dessas iniciativas, não foram identificadas outras experiências nacionais ou internacionais da aplicação do TP em cursos de enfermagem.

Diante do caráter inovador da proposta para o ensino e potenciais benefícios do TP para estudantes, docentes e instituições acadêmicas, propõe-se a reflexão sobre a aplicação unificada do teste em cursos de graduação em enfermagem na perspectiva de repensar as estratégias de avaliação da formação e fomentar a adoção de novas práticas pedagógicas.

## OBJETIVO

Refletir acerca das perspectivas da adoção do TP no ensino de graduação em enfermagem.

## MÉTODOS

Trata-se de estudo reflexivo, baseado no pensamento crítico das autoras e sustentado na literatura nacional e internacional sobre a aplicação do TP, levando em conta as perspectivas do estudante, do docente e da gestão acadêmica.

Tendo em vista o ineditismo dessa iniciativa e a não identificação de literatura nacional ou internacional sobre a aplicação do teste em cursos de graduação em enfermagem, esta reflexão se fundamenta em literatura disponível sobre a aplicação do TP em cursos de graduação na área da saúde.

Esta reflexão está pautada na experiência da aplicação do TP das Escolas de Enfermagem Públicas Paulistas, aplicado pelo Consórcio dos Cursos de Enfermagem Paulistas, composto por oito instituições públicas de ensino do Estado de São Paulo como: EERP-USP; Escola de Enfermagem da USP (EE-USP); EPE-UNIFESP; Faculdade de Medicina de Marília (FAMEMA); Faculdade de Medicina de São José do Rio Preto (FAMERP); Faculdade de Medicina de Botucatu da Universidade Estadual Paulista (UNESP); Faculdade de Enfermagem da Universidade de Campinas (UNICAMP); e Universidade Federal de São Carlos (UFSCar).

O teste é aplicado anualmente, composto por 120 questões objetivas, com distribuição em seis áreas do conhecimento (20 questões de saúde coletiva, 15 de gestão, 20 de saúde da criança e do adolescente, 20 de saúde da mulher, 35 de saúde do adulto e dez de saúde mental), com avaliação de desempenho do estudante mensurada pelo número de acertos e por uma nota em escala de 0 a 10 pontos por área e total.

A elaboração, a revisão e a seleção das questões fazem parte de processo realizado anualmente, conforme relação de temas definidos pelo consórcio e cronograma próprio. As questões são elaboradas por docentes das instituições participantes e revisadas e selecionadas coletivamente por docentes especialistas das áreas, representantes de todas as instituições participantes. Entre as 120 questões do teste, 35 são questões-âncora, que são questões aplicadas anteriormente, distribuídas uniformemente para as seis áreas de conhecimento, que apresentaram ótimo ou bom desempenho psicométrico, enquanto as 85 demais questões são inéditas, elaboradas exclusivamente para o teste do referido ano.

Todos os estudantes de todos os anos das oito instituições de ensino são elegíveis para a realização e incentivados a participar do teste, que tem custos operacionais integralmente cobertos pelas instituições, sem pagamento de qualquer taxa por parte dos estudantes, que participam do teste voluntariamente.

## REFLEXÕES ACERCA DO TESTE DE PROGRESSO NA ENFERMAGEM

As reflexões apresentadas a seguir estão estruturadas desde as perspectivas do estudante, do docente e da gestão acadêmica.

### Estudante

As experiências de aplicação do TP na educação médica apontam para três principais benefícios para os estudantes: avaliação do aprendizado acadêmico; apoio no processo de aprendizagem; e preparo para exames de licenciamento^(6)^.

Para o estudante, o TP possibilita a avaliação periódica de sua evolução ao longo do curso, com reflexão acerca dos conhecimentos obtidos a cada etapa do curso, conformando-se em uma autorresponsabilização por seu aprendizado. Após a realização do teste, o estudante pode analisar seu desempenho por meio do escore obtido (total e por área), da comparação de seu desempenho individual com outros estudantes do seu ano de todas as instituições e com outros estudantes da sua instituição e da magnitude de progressão individual do estudante em relação ao desempenho em testes dos anos anteriores, quando aplicável, sendo possível a reflexão acerca do seu grau de domínio cognitivo nas áreas abordadas no teste^(5)^.

Na perspectiva do *feedback* formativo, além da análise de desempenho, o estudante tem acesso à prova com gabarito comentado e às referências. Para cada questão do teste, são apresentados comentários acerca de itens corretos e incorretos e as referências que subsidiam temas e conceitos abordados em cada uma das questões. Dessa forma, por meio de autogerenciamento e pensamento crítico, o teste possibilita ao estudante a identificação de suas fortalezas e fraquezas e a identificação de oportunidades de melhoria de seu conhecimento.

O TP, por meio de sua devolutiva baseada em gabarito comentado e referências, oferece ao estudante uma oportunidade de aprendizado sobre temas e conceitos esperados para cada área de conhecimento, sendo possível ao estudante buscar melhorar seu desempenho acadêmico e avançar na aprendizagem sobre diversos temas abordados no teste^(3, 5)^.

Tais contribuições são corroboradas pela opinião de estudantes de graduação em medicina que, quando entrevistados sobre sua participação no TP, consideraram o teste uma “ferramenta pedagógica” relevante, que permite a autoavaliação e a correção das lacunas de aprendizagem^(7)^.

Por fim, o TP fornece uma oportunidade para que os estudantes se preparem para a realização de outros exames com estrutura semelhante, como provas de concurso ou processos seletivos de residência. Ainda que não seja uma realidade no Brasil, o TP possibilita o preparo para provas de licenciamento profissional, como a experiência de outros países, como os Estados Unidos da América.

### Docente

Avaliar conhecimentos é uma atividade importante do trabalho docente. Para os docentes, o TP permite reflexão sobre conteúdo abordado em disciplinas e conhecimento esperado para ser adquirido pelo estudante. Com base nos resultados de desempenho dos estudantes, o docente é capaz de realizar um diagnóstico sobre o ensino e a implementação de intervenções educacionais e ações de melhoria contínua do curso, além de identificar oportunidades de desenvolvimento docente.

O TP pode ser utilizado de forma complementar ao modelo tradicional de avaliação de disciplinas ministradas pelos docentes, permitindo análises mais ampliadas do desempenho dos estudantes e/ou das turmas, gerando menor tensão entre docentes e estudantes, quando comparada aos instrumentos de avaliação tradicionais voltados para aprovação/reprovação de estudantes^(4)^.

Ao trabalhar na elaboração, revisão e seleção de questões, o docente pode aprimorar suas habilidades para o desenvolvimento de questões objetivas para avaliação dos estudantes, considerando as boas práticas para elaboração de questões de múltipla escolha. Adicionalmente, é importante destacar a relevância de iniciativas, individuais ou institucionais, para o docente desenvolver a habilidade de elaborar tais questões, uma vez que elas devem garantir que os resultados das avaliações sejam válidos e confiáveis.

Ao trabalhar em conjunto com colegas de outras áreas e outras instituições, o docente pode ampliar a reflexão acerca do perfil de conhecimentos necessários para a formação do enfermeiro, analisando a prática profissional desde a perspectiva de diferentes docentes e instituições.

Nesse sentido, além do suporte na elaboração das questões, os docentes precisam de *feedback* acerca da boa qualidade dos itens, considerando o desempenho dos estudantes e a avaliação psicométrica das questões^(8)^.

### Gestão acadêmica

Do ponto de vista da gestão acadêmica, é possível identificar temas de maior ou menor desempenho, desagregados por ano de ensino e área de conhecimento. Dessa forma, diante da possibilidade de identificar lacunas de formação, é possível propor e implementar ações de melhoria para o curso, tais como adaptar o projeto pedagógico e propor estratégias de ensino-aprendizagem no sentido de garantir aquisição de conhecimento, com vistas a melhorar o ensino.

A análise de desempenho institucional permite realizar inferências acerca da efetividade do currículo do curso, identificando suas fortalezas e vulnerabilidades, com possibilidade de revisá-lo ou mesmo de verificar o impacto de mudanças curriculares realizadas^(5)^.

Assim, tomadores de decisão, como coordenadores de cursos de áreas de ensino e representantes de órgãos colegiados, podem utilizar a avaliação para rever o projeto pedagógico e os conteúdos abordados, além de propor alterações em disciplinas, áreas do conhecimento ou no projeto acadêmico e/ou currículo do curso^(3)^.

Tais aplicações têm sido relatadas na experiência do ensino em escolas médicas, em que o TP tem sido utilizado para avaliar mudanças curriculares, comparar currículos tradicionais e currículos com metodologias ativas de aprendizagem e auxiliar o desenvolvimento de novos currículos^(4, 6)^.

Também é possível realizar comparações (*benchmarking*) desagregadas por área de conhecimento e ano de ensino entre as instituições participantes, permitindo análises contextualizadas, sem ranqueamento, de diferentes estratégias pedagógicas e modelos curriculares^(6)^.

Além das ações mais abrangentes, por meio da análise de desempenho individual dos estudantes, é possível identificar estudantes com alto e baixo desempenho e traçar ações de suporte pedagógico personalizado a cada caso^(6)^.

Diante do exposto, entende-se que as instituições devem assumir papel ativo na operacionalização do TP, incentivando e favorecendo o envolvimento e a participação ativa de estudantes e docentes.

Do ponto de vista institucional, é importante que se invista em medidas que estimulem a participação ativa de todos os estudantes, bem como se fomentem espaços de reflexão e discussão de desempenho entre estudantes e docentes, favorecendo a utilização dos resultados do TP como estratégia de aprendizagem^(5)^.

Cabe à instituição, ainda, promover ações de desenvolvimento docente para a elaboração de questões objetivas, com estratégias como oficinas, orientações técnicas e espaços de discussão sobre a qualidade das questões e a análise dos resultados^(3)^.

### Desafios e limitações

Ainda que haja benefícios na utilização do TP, devem-se considerar suas limitações e desafios. Do ponto de vista operacional, a organização e a realização do TP são tarefas árduas que consideram o envolvimento de diferentes atores e instituições e o estabelecimento de cronograma que se adeque à agenda institucional e à implicação de recursos financeiros^(4)^.

É preciso fomentar discussões entre os atores envolvidos e ter clareza acerca dos objetivos do teste, temas e áreas de interesse, alinhados com as competências requeridas ao enfermeiro, além de estratégias de análise e utilização dos resultados no processo de ensino-aprendizagem^(7)^.

Visando a qualidade e a validade do teste, a capacitação do corpo docente para a elaboração de questões objetivas deve ser considerada em diferentes e contínuas estratégias^(9)^.

Tendo em vista a participação voluntária dos estudantes, é possível inferir que os estudantes participantes são aqueles com maior envolvimento e desempenho no curso. Dessa forma, é importante incentivar a ampla participação de todos os estudantes, de forma a obter resultados que, de fato, representem a instituição como um todo e, consequentemente, contribuam para as ações de melhoria do ensino^(10)^.

Ainda que se considere que o TP possibilita o *feedback* formativo, deve-se considerar que a prática da autoavaliação ainda não se constitui cultura na vida acadêmica, sendo necessário propor espaços de reflexão e discussão de questões e dos resultados individuais, de turmas e do curso^(7)^.

Por fim, cabe ressaltar que o TP proporciona a avaliação de desempenho no nível cognitivo, sendo possível ao estudante obter escore elevado no teste, mas apresentar deficiências no domínio de outras habilidades e atitudes^(3)^. Assim, embora o TP seja uma importante ferramenta, é necessário que seja aplicado de modo integrado com outras estratégias de avaliação para além do nível cognitivo.

## CONSIDERAÇÕES FINAIS

A adoção do TP é uma estratégia de avaliação do ensino de graduação em enfermagem, somando-se às ações para qualificar o processo de ensino-aprendizagem.

A avaliação sistemática do ensino de enfermagem, por meio do TP, permite o fornecimento de informações valiosas para diferentes atores. Considerando os potenciais benefícios do teste para os estudantes, docentes e para a gestão acadêmica, apresenta-se o TP como estratégia passível de ser replicada no ensino de graduação em enfermagem, seja de forma individual, pelas instituições, ou de maneira colaborativa, pelo estabelecimento de parcerias e consórcios entre instituições.
